# Prevalence of Obstructive Sleep Apnea Syndrome: A Single-Center Retrospective Study

**DOI:** 10.3390/ijerph181910277

**Published:** 2021-09-29

**Authors:** Manlio Santilli, Eugenio Manciocchi, Gianmaria D’Addazio, Erica Di Maria, Michele D’Attilio, Beatrice Femminella, Bruna Sinjari

**Affiliations:** 1 Unit of Prosthodontics, Department of Innovative Technologies in Medicine and Dentistry, University “G. d’Annunzio” Chieti-Pescara, 66100 Chieti, Italy; santilliman@gmail.com (M.S.); manciocchieugenio@gmail.com (E.M.); gianmariad@gmail.com (G.D.); beatrice.femminella@yahoo.it (B.F.); 2 Unit of Orthodontics, Department of Innovative Technologies in Medicine and Dentistry, University “G. d’Annunzio” Chieti-Pescara, 66100 Chieti, Italy; erica.dim95@gmail.com (E.D.M.); michele.dattilio@unich.it (M.D.)

**Keywords:** OSAS, dentistry, obstructive sleep apnea syndrome, sleep disorders, sleep medicine

## Abstract

Obstructive sleep apnea syndrome (OSAS) is a sleep breathing disorder that often remains undiagnosed and untreated. OSAS prevalence is increasing exponentially. Starting on the dentist’s role as an epidemiological and diagnostic “sentinel”, the purpose of this study was to assess the prevalence of OSAS. The clinical diaries of 4659 patients were reviewed through a single-center retrospective analytic study. Descriptive statistical analysis was performed. Only 0.26% of patients reported to suffer from sleep apnea and were then diagnosed with OSAS. It was found that, out of 4487 patients, 678 suffered from hypertension (14.80%), 188 from gastro-esophageal-reflux-disease (GERD = 4.10%) and 484 from gastritis (10.78%). These results could be related to a difficult diagnosis of OSAS and to the absence of a dedicated section on sleep disorders in medical records. Therefore, the introduction of a question dedicated to sleep disorders, the administration of questionnaires (such as the STOP-BANG questionnaire) for early diagnosis, a multidisciplinary approach and pneumological examination could support the dentist in identifying patients at risk of OSAS.

## 1. Introduction

Obstructive sleep apnea syndrome (OSAS) is a sleep breathing disorder manifested by complete (apnea) or partial (hypopnea) obstruction of the upper airway, which often remains undiagnosed and untreated [1]. These episodes, which should be more than 5 per hour and last at least 10 s, can lead to a sleep fragmentation and hypoxia [2]. OSAS predominantly affects 26% of individuals between 30 and 70 years in the U.S (apnea-hypopnea index ≥5 events per hour) [3].

A recent study found that out of 38,000 Russian citizens (aged 30–70 years), 48.9% suffered from an AHI (Apnea-Hipopnea Index) ≥ 5, 18.1% from an AHI ≥ 15 and 4.5% from an AHI ≥ 30 [4]. In 2020, Peñafiel et al. observed 205 people (aged between 18–84 years) undergoing overnight respiratory polygraphy and they found a prevalence of 49% AHI ≥ 5 and 16% AHI ≥ 15 [5]. A recent study conducted on 1642 Southern Italian children (aged 6–12 years) showed that the risk of developing an OSAS corresponded to 10.47% [6]. Benjafield et al. found that approximately 936 million adults aged 30 to 69 years have mild to severe OSAS, and 425 million adults aged between 30 and 69 years have moderate to severe OSAS globally, according to the diagnostic criteria of the American Academy of Sleep Medicine (AASM) [7]. Further data analyzing the correlation between the incidence of OSA and age demonstrated that 88% of men aged 65 to 69 years had five or more events per hour, while that incidence increased up to 90% in men aged 70 to 85 years [8]. These data indicate a high presence of this disease, although with different trends. Among the causes there is definitely the use of different diagnostic tools. For the AASM, the gold standard for the diagnosis of this condition still remains comprehensive polysomnography (sleep study), although alternative diagnosis methods have also been studied [9]. Moreover, it has been reported that OSAS represents a risk factor for other diseases, such as hypertension, heart failure, heart attack and cardiovascular event. Indeed, OSAS is related to hypertension and some epidemiological studies suggest that 30–50% of hypertensive patients have OSAS [10,11,12]. Furthermore, a recent meta-analysis states that the risk of essential hypertension was higher with increased AHI values [13]. At the same time, hypertension prevalence was higher (53%) in individuals with severe OSAS, when compared to others having moderate one (46%). Generally, hypertension in OSAS patients showed a prevalence between 30% and 70% [14]. These cardiovascular complications are due in part to a rapid reduction in blood oxygen and to an increase in carbon dioxide concentration [15]. Moreover, a recent meta-analysis compared OSA patients and healthy subjects (by using the Snifn’ Sticks test) and showed lower values of the various olfactory parameters in the first ones [16]. On the other hand, OSAS is strictly correlated also with oral pathology. Indeed, some articles suggested that OSAS may increase pathologies of the hard and soft tissues of the oral cavity. In fact, the snoring that occurs in patients with OSAS can make the oropharyngeal and laryngeal tissues vibrate, thus leading to a release of inflammation mediators. This mechanism increases infiltrating lymphocytes at the soft palate and leads to edema formation, which causes increased upper airway obstruction [17]. A recent study highlighted that OSAS can also be related to a weakening of the hard tooth tissues. In fact, this syndrome is related to gastro-esophageal-reflux disease caused by acid regurgitation in the oral cavity, having similar risk factors such as obesity and gender [18]. Nizam et al. suggest that OSAS has a key role in the development of periodontal inflammation, with bacterial population changes in the subgingival plaque and an increase in salivary concentration of IL-6 and apeline [19]. Consequently, a possible link between periodontitis and OSAS has been hypothesized, although the pathophysiological mechanism and cause–effect still remain unclear [20]. For several years, the dentist has represented an important figure as a diagnostic sentinel, and—along with sleep medicine specialists—had a key role in its therapeutical treatment. Therefore, it is important to underline that OSAS should be addressed in a multidisciplinary way and that teams composed by different figures, such as specialists in pneumology or neurology, maxillofacial specialists, dentists, otolaryngologists and nutritionists, should treat this syndrome in the best possible way [21]. It is also important that the above-mentioned specialists know and fully understand that this condition should be addressed through an appropriate treatment plan. When it comes to the treatment, the “gold standard” remains the use of Continuous Positive Airway Pressure (CPAP). However, this has significant physical and psychological drawbacks such as xerostomia, throat dryness, difficulty on handling such equipment during sleep, nasal obstruction, etc. [22,23]. However, several evidence in the literature reports the need, especially in young subjects, for a tailored OSAS treatment, which is facilitated by innovative methods such as drug-induced sleep endoscopy and robotic surgery [24,25]. In recent years, several intraoral devices have been studied such as Mandibular Advancement Device (MAD). These devices play a key role in the treatment of OSAS. In fact, MAD moves the jaw forward, greatly increasing the volume of the upper airways and then representing the device chosen for those patients having moderate to severe OSAS and not accepting CPAP (which is certainly more invasive and discomforting) nor surgery treatment [26]. Moreover, MAD’s design is customized, adapted and definitely cheaper than CPAP [27]. Both CPAP and oral devices lead to an increase in the quality of life of patients and they could have effect on minimizing apnea–hypopnea events, oxygen desaturation, sleepiness and on reducing therapeutic pressure [26,27]. However, the decision on the use and characteristics of the type of oral devices to be used is a dentist’s responsibility [26,27]. Unfortunately, not all dentists are familiar with the diagnosis or treatment of this pathology. In fact, it would be appropriate to prepare dentists throughout their education, to let them serve as a bridge between patients and sleep medicine specialists [28]. In dentistry, medical records often do not allow for easy framing of the patient from a systemic perspective. In fact, the medical records are usually completed by patients or their parents without relying on health care providers [29]. Patients also think it is irrelevant for dental purposes to inform their dentist about the condition of systemic diseases. Furthermore, there is also inconsistency among professionals about how a patient’s medical history is recorded. Even the terms listed in the medical record can be a source of misunderstanding, such as hypertension or high blood pressure [30]. Therefore, there is often no specific question for sleep disorders in medical record questions, which leads both patients and clinicians to a lack of understanding of the actual state of health. To the best of our knowledge, there were no studies that addressed the prevalence of OSAS in the dental population. So, starting from the epidemiological and diagnostic “sentinel” role of the dentist, the purpose of this article was to evaluate, through a retrospective investigation of medical records, the prevalence of OSAS without a diagnostic tool and assess how important it was to implement the diagnostic pathway by dentists. The secondary outcome of this study was to evaluate the correlation of OSAS with other systemic diseases.

## 2. Materials and Methods

### 2.1. Study Design and Data Collection

This study has been carried out following the STROBE guidelines specified for this retrospective single-center study. The authors reviewed the clinical diaries of 4659 patients treated in the dental clinic of the Department of Innovative Technologies in Medicine and Dentistry of “G. Annunzio University” of Chieti and Pescara, in the period from January 2016 to January 2020. All patients included had undergone at least an initial dental examination and a first-level radiological examination (orthopantomography). Specifically, the initial dental examination was performed by analyzing general medical history, dental history, intra- and extra-oral examination, and X-ray inspection. Data were collected through a medical record provided by ANOE (National Association of Ethical Dentistry) [31]. Male and female patients aged 7 to 89 years were considered. Patients without complete data were excluded. Therefore, 4659 medical records were initially considered and 4487 were subsequently included. An Excel worksheet (Microsoft Corporation, Redmond, WA, USA) was created in which gender, age, respiratory disease, OSAS, general systemic disease, drugs and smoking behaviour were reported.

### 2.2. Ethical Consideration

Participants provided their written informed consent to the processing of personal data in accordance with the 95/46/CE directive and the EU General Data Protection Regulation GDPR (UE) n. 2016/679. Data collection took place in the time period from 1 June 2020 to 31 March 2021. The study was approved by the Ethic committee of the “G.d’annunzio” University with approval number 15 of 17 June 2021.

### 2.3. Statistical Analysis

#### 2.3.1. Sample Size Calculation

In accordance with the relevant literature [32,33], OSAS tends to have a high prevalence affecting 26% of individuals aged 30–70 years in the USA. Based on the percentages in the literature, a sample size of 578 subjects was calculated to have, at the end of the retrospective study, a possible statistically significant difference between the test subjects and the general population. The value of α was determined at 0.05 while the Power of the test was at 0.80. Considering the possible causes of exclusion described, an increase of 10% was inserted. In this way, the minimum number of folders to be analyzed was 636. The https://clincalc.com/stats/samplesize.aspx (accessed on 15 July 2021)website was used for the calculation [34].

#### 2.3.2. Statistical Analysis

Some of the answers were codified as dichotomous variables, namely as Yes/No responses, or in general as categorical variables, when a multiple-choice selection was requested. Given the nature of this retrospective study, the authors performed descriptive statistics for all medical data in the medical record. For each datum, the percentage of respondents with a positive anamnesis was recorded. The statistical analyses were performed using the GraphPad version 8 (GraphPad Software 2365 Northsides Dr. Suite 560 San Diego, CA 92108) statistical software [35].

## 3. Results

**Figure 1:**
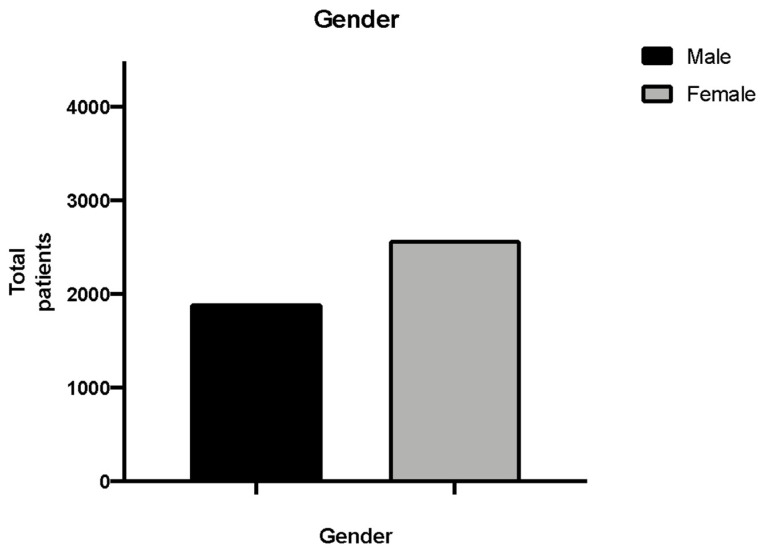
Gender distribution.

Out of 4659 clinical folders selected, 4487 were included in the study. A total of 172 were considered as drop-outs since they lacked data or were incomplete. The results demonstrated that 1903 of the clinical folders included in this retrospective study belonged to men and 2584 to women, respectively 42.21% and 57.8% of the total, as shown in Figure 1.

**Figure 2:**
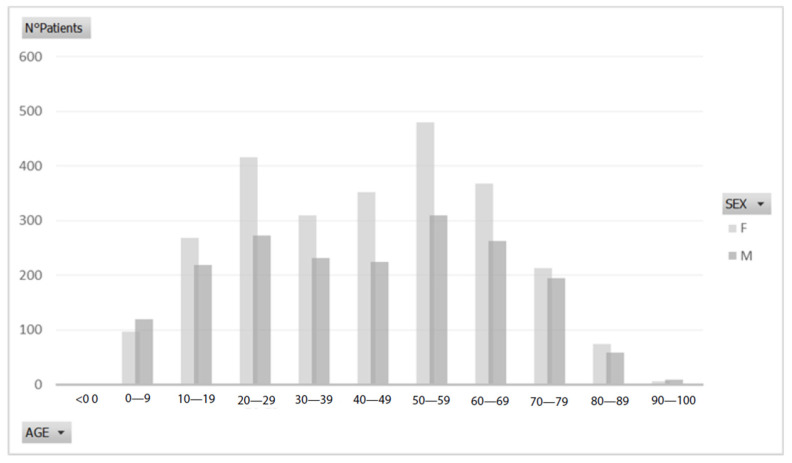
Demographic data.

The age range of the patients was from 7 to 89 years. The mean age of patients was 43.6 years (Figure 2).

**Figure 3:**
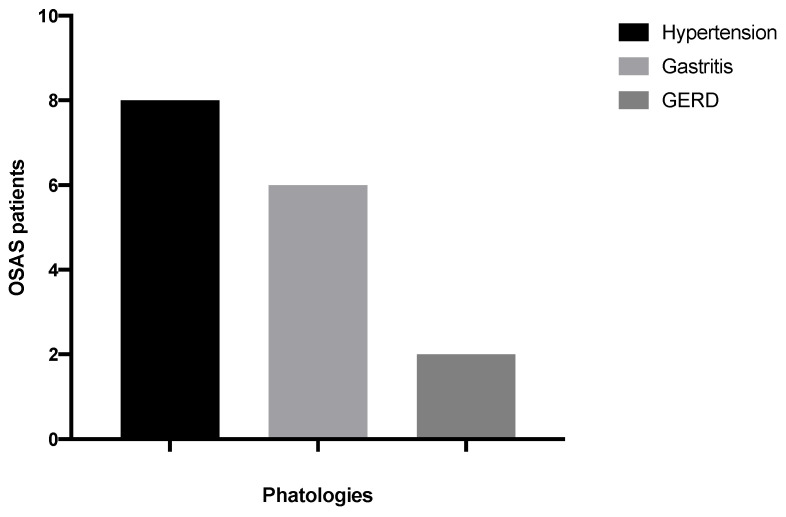
Distribution of pathologies in OSAS patients.

Only 12 (9 men and 3 women) (0.26%) reported suffering from sleep apnea and were diagnosed with OSAS by a sleep medicine specialist in the past. Out of these, 8 patients (5 men and 3 women) had hypertension, 6 had gastritis (3 men and 3 women), and 2 had gastro-esophageal-reflux-disease (1 man and 1 woman) (Figure 3). Only 2 patients referred not to take medications for their systemic disease, thus they were uncontrolled. Out of 12 patients, 3 were smokers, 7 non-smokers and 2 were former smokers. Only 3 patients benefit from CPAP use, whereas the other 9 do not use medical devices.

**Figure 4:**
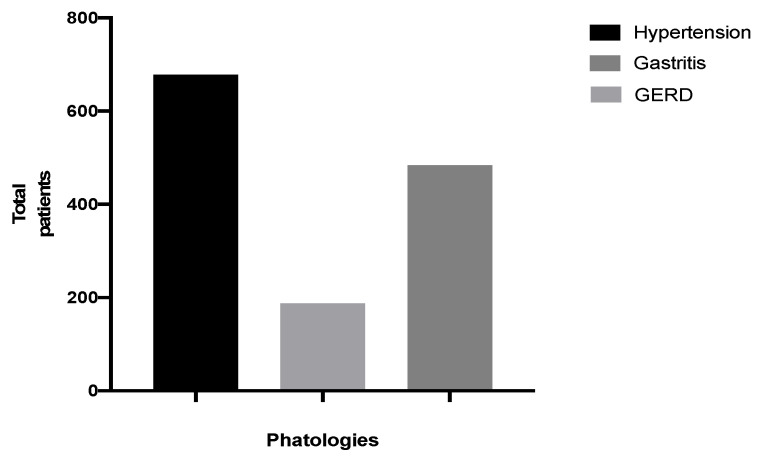
Distribution of systemic pathologies in patients.

Data on major systemic diseases recorded during their medical history were collected from all patients. Furthermore, out of 4487 patients, 678 were found to suffer from hypertension (14.80%), 188 from GERD (4.10%) and 484 from gastritis (10.78%) (Figure 4).

**Figure 5:**
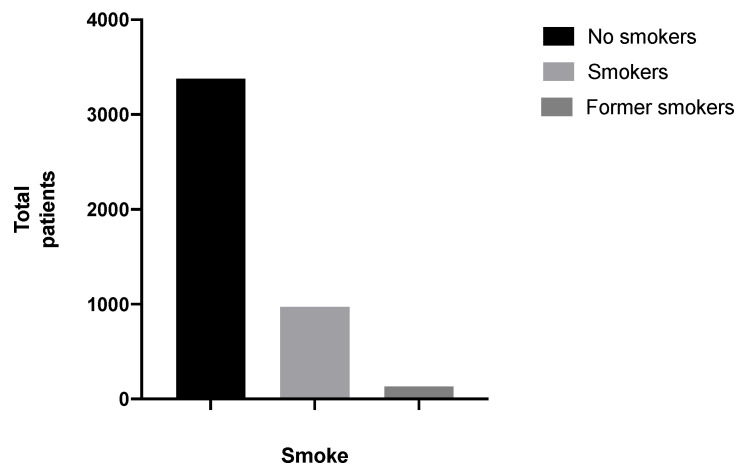
Smokers, non-smokers and former smokers.

Among 4487 patients, there were smokers, non-smokers and ex-smokers, respectively, 973 (21.68%), 3377 (75.26%) and 133 (2.96%). The remaining four patients did not report these data in the medical record (0.09%) (Figure 5).

## 4. Discussion

The literature states that the prevalence of OSAS in the general population is approximately 13% for men and 6% for women [36]. In addition, the prevalence of OSAS in the general adult population varies between 9% and 38%, but approximately 80–90% of OSAS cases remain undiagnosed [37]. In the present study, the percentage of patients with OSAS drops dramatically to 0.26%. This decrease could be related to the difficult diagnosis of this syndrome and the absence of a dedicated section on sleep disorders in the medical records used in the present study. In addition, patients having episodes of respiratory distress overnight do not have respiratory abnormalities during wakefulness [38], which leads them not to consult a specialist. In fact, one of the most common symptoms during the day is daytime sleepiness, which has little diagnostic value for most people [39]. The gold standard for the diagnosis of OSAS still remain polysomnography [9], even if other alternatives have also been studied such as a home sleep apnea test (HST) with pulse-oximetry. In fact, even if some alternative tests such as the HST could be considered a cost-effective solution for the diagnosis of OSAS, it has been demonstrated that they are not recommended for the definitive diagnosis of this syndrome [40,41]. On the other hand, it should be noted that polysomnography is very expensive and must be performed for at least two nights in a row. In addition, the waiting list can be up to 1 year in some cases [42], and in order to perform a proper sleep analysis, it is necessary to sleep in a clinic or hospital. In addition, this type of examination requires the constant presence of a sleep technician. Ultimately, the main problem associated with polysomnography is that this is not considered suitable for long-term sleep monitoring [43]. Approximately 50% of general practitioners do not screen people at high risk for OSAS and 90% of these do not use OSAS screening tools [44]. What emerges from the present study is the low prevalence of OSAS. As mentioned above, this could be caused by the fact that OSAS is a difficult disorder to be diagnosed also because it is quite common that patients are not aware that they suffer from this syndrome. Other authors have evaluated the real effectiveness of medical records in diagnosing other pathologies. Adibi et al. noticed that 15.1% of patients do not report diabetes and 29% do not report high blood pressure [30] when the question is not well addressed or is missing. According to the authors, this latter aspect has played a crucial role in this study’s results as well. It has been observed that a medical record which is not accompanied by specific questions about sleep disorders very rarely leads the patient to voluntarily report them. Although the medical record we use is accredited by ANOE [31], the authors think that the low prevalence detected is also due to the lack of specific questions about sleep disorders. In the literature, among the various diagnostic tools used, the STOP-BANG (Snoring, Tiredness, Observed apnea, blood Pressure, Body mass index, Age, Neck circumference and Gender) seems to be the most effective. In fact, the STOP-BANG questionnaire has been shown to be a valid screening tool. A score of 3 has a sensitivity between 88% and 92%, and identifies those who are at risk for moderate to severe OSAS [37]. In addition, the sensitivity of the STOP-BANG questionnaire is higher than that of the Berlin questionnaire, the Epworth scale and the STOP questionnaire for identifying mild, moderate or severe OSAS [45]. Thus, given the score obtained from the STOP-BANG questionnaire, patients can be used as screening for OSAS. In fact, patients with a score of 0 to 2 can be classified at low risk of moderate to severe OSAS, while patients with a score from 5 to 8 at high risk of moderate to severe OSAS [46]. Since the STOP-BANG questionnaire is a valuable tool for assessing OSAS risk, it would be desirable to include it in the diagnostic pathway of dental patients. Moreover, this questionnaire is simple and intuitive for patients and can be completed in less than 2 min [47]. It is to be considered that in recent years, OSAS has also been recognized as a medical condition in children, with a prevalence of 1% to 5% [48,49]. Therefore, the role of the dentist and orthodontist is definitely crucial, as there may be clinical conditions that could increase the risk of OSAS. In fact, anomalies of the jaws, such as micrognathism and retrognathism, or of the soft tissues, such as macroglossia, can lead to a reduction in oropharyngeal space [50]. Considering that dental patients get routine checkups, the use of a questionnaire for OSAS can certainly help to diagnose this disease earlier and implement further evaluations [51]. In addition, it has been shown that patients are well disposed to screening in dental offices and that also dentists are willing to perform screening tests [52,53]. Literature has shown that the use of the STOP-BANG questionnaire in dentistry can indeed help patients in their diagnostic process. In fact, Lonia et al. saw that 482 of 1000 patients recruited were at risk of OSAS after completing the STOP-BANG questionnaire, and among these, 121 received an OSAS diagnosis after taking a type 3 polygraphy [54]. Also for these reasons, it would be advisable to integrate a specific question about sleep disorders as well as a questionnaire into the medical record, since it can greatly support the identification of this under-diagnosed condition. Moreover, this study also shows how some diseases are closely related to OSAS. In fact, out of 12 OSAS patients, 66.6% suffered from hypertension showing that OSAS and hypertension are closely related. The literature shows that 50% of OSAS patients suffer from hypertension and 30% of hypertensive patients suffer from OSAS [55]. In addition, the results of a meta-analysis conducted have concluded that there is an association between OSAS and hypertension, showing that an increase of OSAS severity increases also the risk of hypertension [13]. Gastroesophageal reflux is also considered one of the potential causes of OSAS. In fact, these two conditions can often coexist [56]. Furthermore, one study showed that GERD is much more frequent in patients with OSAS than in a control group (43.3% versus 13.3%) [57]. In addition, Basoglu et al. demonstrated that 38.9% of patients with OSAS showed GERD, against non-OSAS patients who showed GERD in 32.0% of cases. The same authors state that GERD is found in patients regardless of the severity of OSAS [57].

### Study Limitations

The present study’s results showed that on the whole of medical records evaluated, only 4.10% reported the presence of GERD. Out of 12 OSAS patients, two of them reported suffering from GERD. First of all, being a retrospective study, it is not possible to correlate the effectiveness of additional diagnostic tools on the same patients. Based on the difficulties in diagnosing OSAS, the present study found even lower data than the one reported in literature, corresponding to a prevalence of 0.26%. Moreover, not having performed a specific questionnaire for the OSAS may lead to unrevealed data. Thus, it will be a future goal of the authors to administer a questionnaire to the same patients (4487) considered in the present study in order to assess how the prevalence of OSAS may change.

## 5. Conclusions

OSAS remains a difficult group of conditions to diagnose. This is due to the fact that patients are not able to discriminate between a healthy condition and a mild degree of OSAS (AHI > 5). They are not treated by a specialist until they have a feeling of discomfort. Furthermore, OSAS is an underdiagnosed pathology also because patients themselves underestimate the symptoms because they are not always so evident. Therefore, it is likely that the prevalence of OSAS in the literature is underestimated. For these reasons, it would be necessary to introduce in dental records a question and questionnaires specifically dedicated to sleep disorders, which can help the clinician in the identification of patients at risk of OSAS.
